# 2B9-Fc Ameliorates Immune Thrombocytopenia by Suppressing STAT1-Mediated M1 Macrophage Polarization and Expanding Regulatory T Cells

**DOI:** 10.3390/ijms27219658

**Published:** 2026-10-28

**Authors:** Shuran Yang, Shuang Xing, Xing Shen, Wanlu Zhu, Mengting Ge, He Xiao, Kaikai Yu, Zuyin Yu

**Affiliations:** 1 Hengyang Medical College, University of South China, Hengyang 421001, China; yangshuran0913@163.com; 2 Academy of Military Medical Sciences, Beijing 100850, China; xshuang6564@163.com (S.X.); shen_xingjyk@163.com (X.S.); 19855256852@163.com (M.G.); 3 State Key Laboratory of National Security Specially Needed Medicines, Beijing 100850, China; zhuwanllu@163.com (W.Z.); xiaoheysy@163.com (H.X.); 4 College of Chemistry and Life Science, Beijing University of Technology, Beijing 100124, China; 5 School of Pharmacy, Bengbu Medical University, Bengbu 233030, China

**Keywords:** immune thrombocytopenia, thrombopoietin receptor agonist, macrophage polarization, regulatory T cells, c-MPL receptor

## Abstract

Immune thrombocytopenia (ITP) is an autoimmune bleeding disorder in which enhanced platelet destruction and impaired platelet production coexist, and a substantial proportion of patients remain refractory to available therapies. Here, we evaluated 2B9-Fc, a thrombopoietin (TPO) mimetic peptide engineered from a cyclic peptide with high affinity for both human and mouse c-MPL, in passive (anti-CD41 monoclonal antibody R300) and active (guinea pig anti-mouse platelet serum, GP-APS) immunization male C57BL/6J and humanized c-MPL receptor transgenic mouse models of ITP. 2B9-Fc (500 μg/kg, subcutaneously) significantly elevated peripheral platelet counts in both models (*p* < 0.01), without affecting WBC, RBC, or hemoglobin, and alleviated skin purpura and bleeding scores in the active model. In addition, 2B9-Fc treatment was associated with a reduced splenic M1/M2 macrophage ratio, increased regulatory T cells (Tregs), downregulated IL-6 and iNOS while upregulating TGF-β1 and Foxp3, and suppressed STAT1 phosphorylation. In humanized c-MPL receptor transgenic (MT) mice, 2B9-Fc promoted CD41^+^ megakaryocyte generation in vitro and sustained platelet recovery in vivo through day 14. These results indicate that 2B9-Fc ameliorates ITP and is associated with promotion of megakaryocyte maturation and restoration of immune tolerance, likely involving inhibition of STAT1-mediated M1 polarization and Treg expansion, supporting its potential for clinical translation.

## 1. Introduction

Immune thrombocytopenia (ITP) is an autoimmune bleeding disorder defined by isolated thrombocytopenia (<100 × 10^9^/L). Its pathogenesis involves both enhanced platelet destruction and inadequate platelet production [1,2,3]. Conventional therapies, including corticosteroids, intravenous immunoglobulin (IVIG), and splenectomy, fail to induce adequate or durable responses in many patients, and refractory ITP remains a clinical challenge [4,5]. Thrombopoietin receptor agonists (TPO-RAs), such as recombinant human thrombopoietin (rhTPO), eltrombopag, avatrombopag, and romiplostim, are now established as second-line therapy by activating the c-MPL receptor to promote megakaryocyte differentiation and platelet production [6,7,8]. Nevertheless, approximately 20–30% of patients with chronic ITP respond inadequately to or cannot tolerate existing TPO-RAs, and small-molecule TPO-RAs lack activity in rodents and other non-human primates, restricting preclinical evaluation [9,10].

TPO is the key cytokine regulating megakaryocyte development and platelet production. TPO binding to c-MPL on hematopoietic stem/progenitor cells and megakaryocytes [11] activates downstream signaling, including JAK2/STAT5 and PI3K/AKT, and thereby promotes cell survival, proliferation, and differentiation [12,13,14,15]. TPO mimetic peptides offer low immunogenicity, structural simplicity, and ease of genetic engineering. Romiplostim, the first approved TPO mimetic peptide–Fc fusion protein (peptibody), shares no sequence homology with endogenous TPO, reducing the risk of neutralizing antibodies [16]. However, most TPO mimetic peptides reported to date were selected against the murine c-MPL receptor and show insufficient affinity for human c-MPL, which limits clinical translation [17].

Previously, using the reported murine c-MPL-binding peptide AF12193 (GGCADGPTLREWISFCGG) [18] as a scaffold, we screened a randomly mutated phage-display peptide library and isolated a cyclic peptide, 2B9, with high affinity for both human and mouse c-MPL [19]. Fusing 2B9 to the Fc fragment of human IgG1 yielded the long-acting TPO mimetic peptide 2B9-Fc. Here we evaluated 2B9-Fc in passive and active immunization ITP mouse models and examined its mechanisms, focusing on immune regulation (macrophage polarization, Tregs, and DCs) and megakaryocyte generation. We further used humanized c-MPL receptor transgenic mice to verify that 2B9-Fc activates the human receptor, providing a basis for clinical translation.

## 2. Results

### 2.1. B9-Fc Elevates Peripheral Platelet Counts and Hematologic Parameters in the Passive Immunization ITP Model

**Figure 1:**
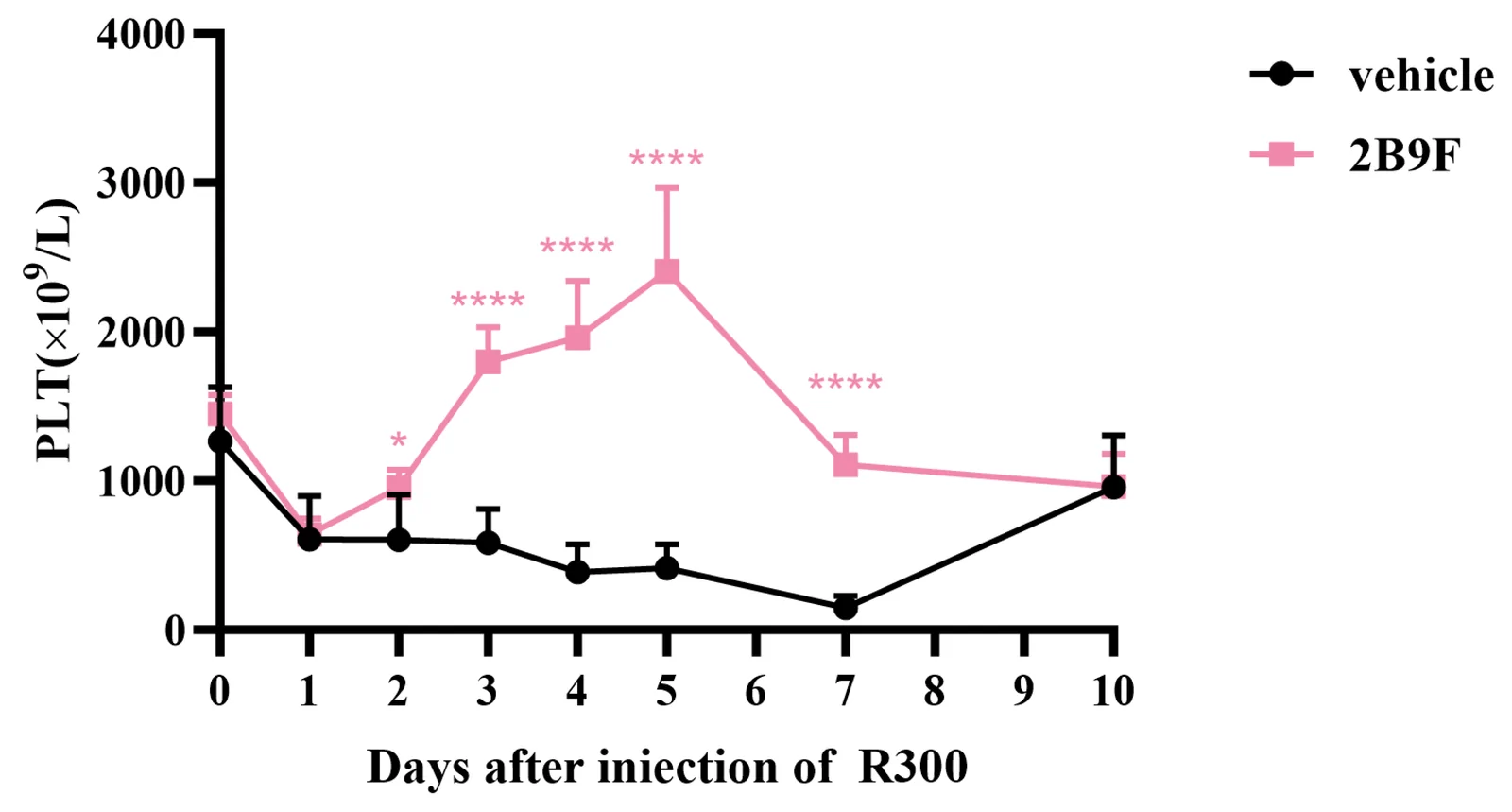
Peripheral platelet counts in the passive immunization ITP mouse model. Dynamic changes in peripheral PLT. 2B9-Fc significantly increased PLT compared with the vehicle group. No significant changes in WBC, RBC, or HGB were observed between groups (*p* > 0.05; Figure S1). n = 6 per group. * *p* < 0.05, **** *p* < 0.0001.

To evaluate the thrombopoietic effect of 2B9-Fc, a passive immunization ITP model was established with the anti-CD41 monoclonal antibody R300, and peripheral blood parameters were monitored dynamically. At 24 h after R300 injection, PLT fell below 650 × 10^9^/L in both the model and treatment groups, with no significant difference between them; PLT in the model group remained low for 7 days, confirming successful modeling. From day 2, PLT in the 2B9-Fc group recovered rapidly and reached a peak of 2403 ± 561 × 10^9^/L on day 5, significantly higher than the model control at the same time point (414 ± 162 × 10^9^/L; mean difference 1989 × 10^9^/L, 95% CI 1402 to 2577; Hedges’ g = 4.45, 95% CI 2.38 to 6.51; *p* < 0.001); the treatment group remained significantly higher on days 3 through 7 (Figure 1). After day 7, PLT in the model group recovered spontaneously as the passively transferred anti-CD41 antibody was gradually cleared. On day 10, PLT counts in the 2B9-Fc group were no longer significantly different from those in the model group (*p* > 0.05), consistent with the known kinetics of this model. Thus, 2B9-Fc rapidly initiated platelet regeneration and sustained a strong platelet-enhancing effect during the acute period of antibody exposure.

2B9-Fc treatment did not significantly affect WBC, RBC, or HGB (*p* > 0.05), indicating selective action on the megakaryocyte lineage without disturbing erythroid or myeloid homeostasis (Figure S1). Given this rapid thrombopoietic effect and the absence of direct interference with peripheral immune cells, the immunomodulatory mechanisms of 2B9-Fc were further explored in both models. Despite the marked reduction in PLT, no obvious skin purpura or bleeding manifestations were observed in the passive immunization model.

### 2.2. B9-Fc Modulates Immune Cell Composition in the Bone Marrow and Spleen of the Passive Immunization ITP Model

**Figure 2:**
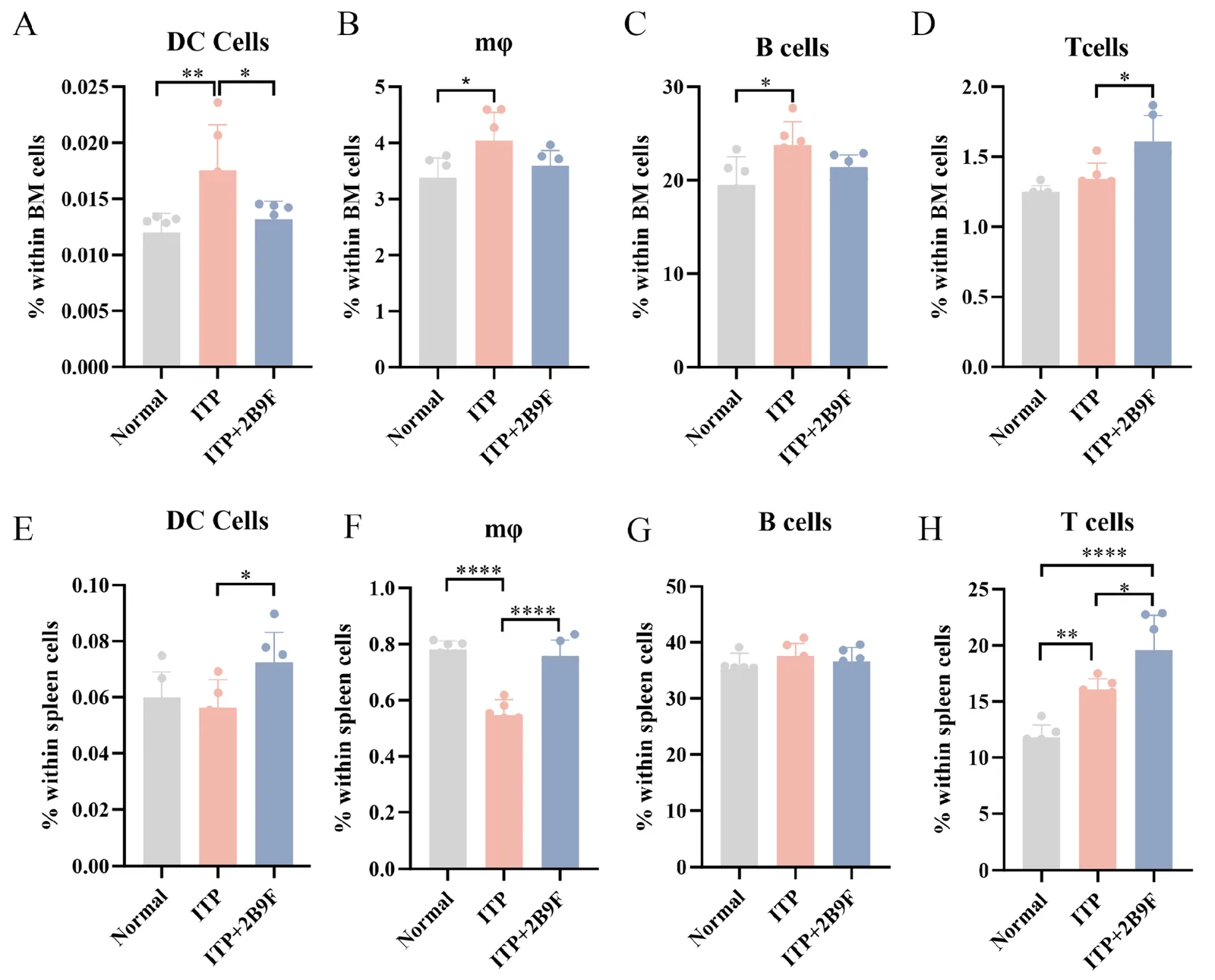
Immune cell composition in the bone marrow and spleen of the passive immunization ITP mouse model. (**A**–**D**) Changes in the composition of major immune cell populations in the bone marrow ((**A**): DCs; (**B**): macrophages; (**C**): B cells; (**D**): T cells); (**E**–**H**) changes in the composition of major immune cell populations in the spleen ((**E**): DCs; (**F**): macrophages; (**G**): B cells; (**H**): T cells). n = 6 per group. * *p* < 0.05, ** *p* < 0.01, **** *p* < 0.0001.

To explore how 2B9-Fc ameliorates thrombocytopenia, we used flow cytometry to profile major immune cell populations in the bone marrow and spleen of the passive model. In the bone marrow, DC, macrophage, and B-cell proportions were all elevated in the ITP group relative to the normal group (*p* < 0.05). 2B9-Fc restored all three populations to normal levels and increased the total T-cell proportion (*p* < 0.05), suggesting that the drug resolved aberrant local immune activation and restored hematopoietic microenvironment homeostasis (Figure 2A–D, Figures S2 and S3).

In the spleen, the composition shifted in the opposite direction. Macrophage proportions were reduced and T-cell proportions increased in the model group. 2B9-Fc significantly recovered DC and macrophage proportions relative to the model group and further expanded T cells (*p* < 0.05), while B-cell proportions did not differ among groups (Figure 2E–H). Thus, 2B9-Fc may participate in immune remodeling in the spleen, the major immune effector organ, possibly by recruiting or inducing specific DC and macrophage populations.

2B9-Fc thus regulated the bone marrow and spleen in an organ-specific manner. Because splenic T cells and macrophages responded strongly to the drug and are closely linked to the loss of immune tolerance in ITP [20,21], we next examined Treg differentiation and macrophage M1/M2 polarization in the active immunization model.

### 2.3. B9-Fc Alleviates Thrombocytopenia and Skin Purpura in the Active Immunization ITP Model

**Figure 3:**
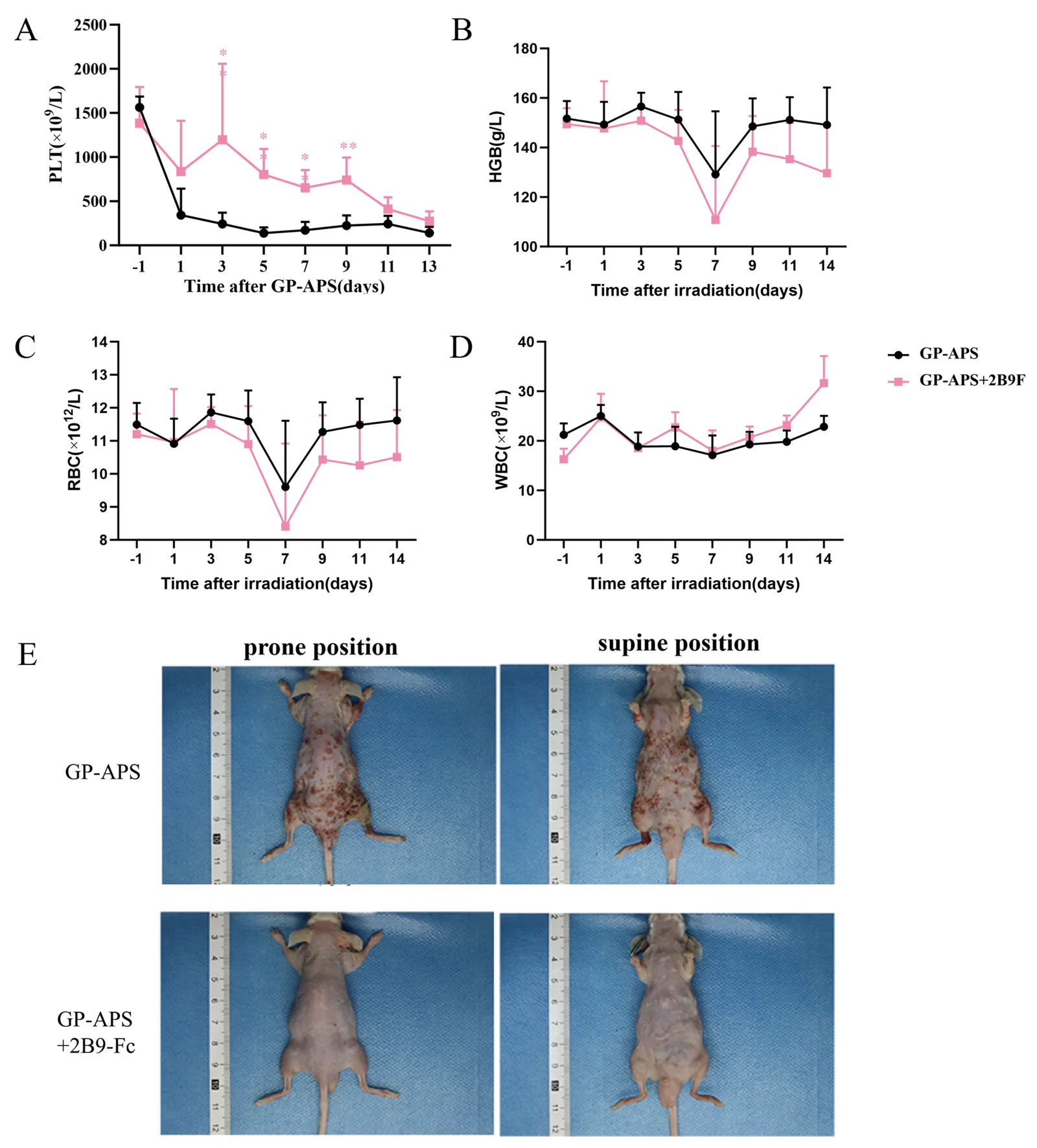
Peripheral blood parameters and skin purpura in the active immunization ITP mouse model. (**A**–**D**) Changes in peripheral blood parameters after 2B9-Fc administration in the active immunization ITP model ((**B**): HGB, (**C**): RBC, (**D**): WBC). (**E**) Representative images of skin purpura in GP-APS and GP-APS + 2B9-Fc mice at day 13, showing supine and prone positions. * *p* < 0.05, ** *p* < 0.01.

To test 2B9-Fc against autoantibody-mediated chronic ITP-like pathology, we established an active immunization ITP model by intraperitoneal injection of GP-APS and monitored platelet counts and bleeding manifestations. After GP-APS injection, PLT in the model group declined progressively, reached a nadir on day 5, and stayed low throughout monitoring. In the 2B9-Fc group (500 μg/kg, subcutaneously, once every 4 days), the decline was markedly limited: recovery began on day 3, and PLT remained significantly higher than in the model group from day 1 onward (*p* < 0.05 or *p* < 0.01) until the end of the experiment (Figure 3A). 2B9-Fc therefore antagonized sustained antibody-mediated platelet destruction and accelerated platelet regeneration. To determine whether 2B9-Fc selectively affects the megakaryocyte lineage in the active immunization model, we also measured WBC, RBC, and HGB. No significant differences were observed among the normal, ITP, and ITP + 2B9-Fc groups for WBC, RBC, or HGB (*p* > 0.05; Figure 3B–D). These results are consistent with the passive model and support a selective effect of 2B9-Fc on platelet production without disturbing erythroid or myeloid homeostasis.

Consistent with the platelet dynamics, model mice developed scattered petechiae and patchy purpura on the dorsal and limb skin on days 5–7, and some showed paw pad congestion, indicating an active bleeding tendency. In the 2B9-Fc group, the area and number of purpura were significantly reduced, with only mild subcutaneous petechiae in individual mice (Figure 3E and Figure S4). Thus, 2B9-Fc not only maintained platelet levels during acute antibody-driven destruction but also relieved bleeding complications caused by persistent autoantibodies, suggesting therapeutic effects beyond the mere promotion of platelet production, possibly involving broader immune homeostasis. We therefore focused subsequent experiments on Treg differentiation and macrophage M1/M2 polarization in the active model.

### 2.4. B9-Fc Promotes Treg Differentiation and Modulates Macrophage Polarization in the Active Immunization ITP Model

**Figure 4:**
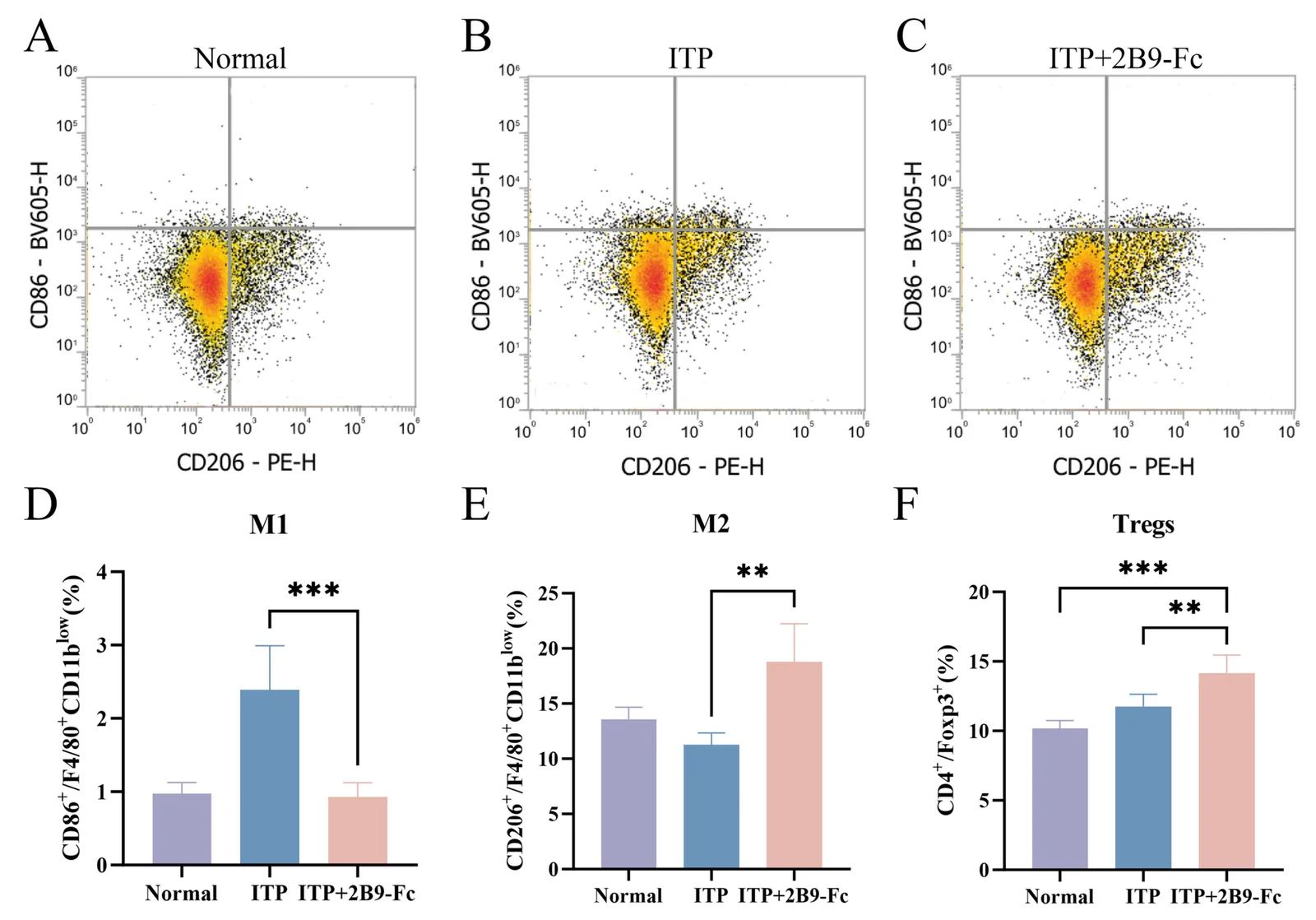
Macrophage polarization and Treg subsets in the active immunization ITP mouse model. (**A**–**C**) Representative proportions of splenic macrophages in the active immunization ITP model ((**A**): Normal group; (**B**): ITP group; (**C**): ITP + 2B9-Fc group); Different colors indicate the gated M1 (CD11b^+^F4/80^+^CD86^+^) and M2 (CD11b^+^F4/80^+^CD206^+^) macrophage populations. (**D**) proportion of splenic M1 macrophages; (**E**) proportion of splenic M2 macrophages; (**F**) proportion of splenic Tregs. n = 6 per group. ** *p* < 0.01, *** *p* < 0.001.

Flow cytometry showed that the splenic M1 macrophage proportion (CD11b^+^F4/80^+^CD86^+^) was significantly higher, and the M2 proportion (CD11b^+^F4/80^+^CD206^+^) significantly lower, in the active ITP model than in the normal group, yielding a markedly elevated M1/M2 ratio (*p* < 0.01) and a pro-inflammatory splenic microenvironment. After 2B9-Fc, the M1 proportion fell (adjusted *p* < 0.001), the M2 proportion recovered (adjusted *p* = 0.001), and the M1/M2 ratio was significantly reduced (0.212 ± 0.050 in the model group vs. 0.052 ± 0.019 after treatment; mean difference −0.160, 95% CI −0.219 to −0.102; Hedges’ g = −3.87, 95% CI −5.90 to −1.83; adjusted *p* < 0.001), indicating reversal toward the M2 phenotype and a reparative local environment (Figure 4A–E and Figure S5).

The splenic CD4^+^CD25^+^Foxp3^+^ Treg proportion was already compensatorily elevated in the model group relative to the normal group (*p* < 0.05), suggesting negative-feedback regulation in response to sustained autoantibody attack. 2B9-Fc further increased the Treg proportion relative to the model group (*p* < 0.01), indicating that the drug exceeded the limited compensatory capacity of the host, actively expanding or inducing Treg differentiation and strengthening immune tolerance (Figure 4F and Figure S6). Together, these results indicate that 2B9-Fc re-established splenic immune microenvironment homeostasis through dual-directional regulation: inducing M2 macrophage polarization and promoting Treg differentiation and expansion. This may constitute an important cellular basis for the control of thrombocytopenia and bleeding in the active model. Accordingly, the expression of relevant cytokines was examined in subsequent experiments.

### 2.5. Effects of 2B9-Fc on Splenic M1/M2 Polarization-Related Gene Expression and the STAT1 Signaling Pathway

To verify the transcriptional effects of 2B9-Fc on the splenic microenvironment, we measured the splenic expression of M1-associated (TNF-α, IL-6, iNOS) and M2/Treg-associated (Arg-1, TGF-β1, Foxp3) genes by qPCR.

**Figure 5:**
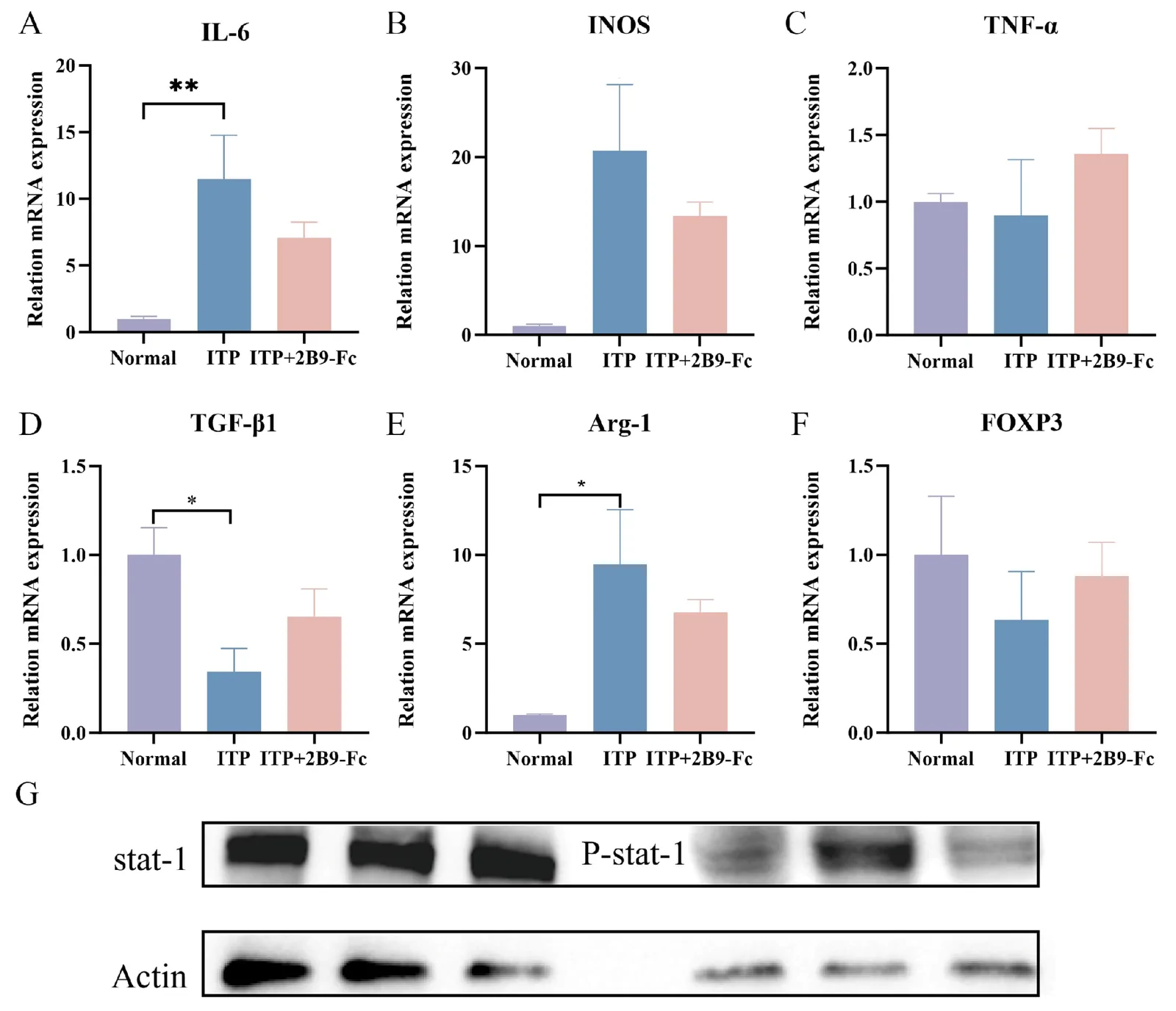
M1/M2 polarization-related gene expression and STAT1 signaling pathway activation in the active immunization ITP model. (**A**–**F**) Expression of M1/M2 polarization-related genes in the active immunization ITP model ((**A**): IL-6; (**B**): iNOS; (**C**): TNF-α; (**D**): TGF-β1; (**E**): Arg-1; (**F**): Foxp3). (**G**) STAT1 signaling pathway activation assessed by Western blotting. n = 6 per group. *p* values for the qPCR data were additionally adjusted across genes using the Benjamini–Hochberg procedure. * *p* < 0.05, ** *p* < 0.01.

Among M1-associated pro-inflammatory factors, splenic IL-6 mRNA were significantly elevated in the model group (q = 0.031) and iNOS showed a consistent upward trend that did not reach significance after FDR adjustment (q = 0.113); TNF-α did not differ among groups (*p* > 0.05). 2B9-Fc treatment was associated with suppression of the M1-associated inflammatory transcriptional program in the spleen (Figure 5A–C). Among M2/Treg-associated effectors, splenic TGF-β1 and Foxp3 mRNA were significantly decreased in the model group (*p* < 0.01) and significantly recovered after treatment (*p* < 0.05). Arg-1 was abnormally elevated in the model group (*p* < 0.01) and, although reduced by treatment, remained higher than in the normal group (*p* < 0.05) (Figure 5D–F).

Because STAT1 signaling is a core transcriptional hub for M1 polarization, we examined total STAT1 and Tyr701-phosphorylated STAT1 (p-STAT1) in splenic tissue by Western blotting. The p-STAT1/STAT1 ratio was significantly elevated in the model group (*p* < 0.01), whereas total STAT1 did not differ among groups (*p* > 0.05), indicating that STAT1 activation occurred mainly at the level of phosphorylation. 2B9-Fc significantly reduced the p-STAT1/STAT1 ratio relative to the model group (*p* < 0.05), toward normal levels (Figure 5G), indicating suppression of aberrant STAT1 activation in the ITP spleen.

### 2.6. B9-Fc Activates the Humanized c-MPL Receptor and Elevates Hematologic Parameters in the Humanized ITP Model

The experiments above confirmed immunomodulatory and hematopoietic effects in wild-type mice, but whether 2B9-Fc directly activates the human c-MPL receptor remained to be established. We therefore cultured bone marrow cells from humanized c-MPL receptor transgenic (MT) mice in vitro and established an active immunization ITP model in MT mice for in vivo evaluation. MT mice were generated by gene replacement at the endogenous Mpl locus. Sequencing confirmed the correct humanized sequence, and all mice used were homozygous (Figure S7A). qPCR showed that Mpl mRNA expression in sorted LSK cells and Lin^−^CD41^+^ megakaryocytic lineage cells was comparable between WT and MT mice (*p* > 0.05; Figure S7B,C), indicating that the gene replacement did not alter endogenous Mpl expression levels or cell-type distribution.

**Figure 6:**
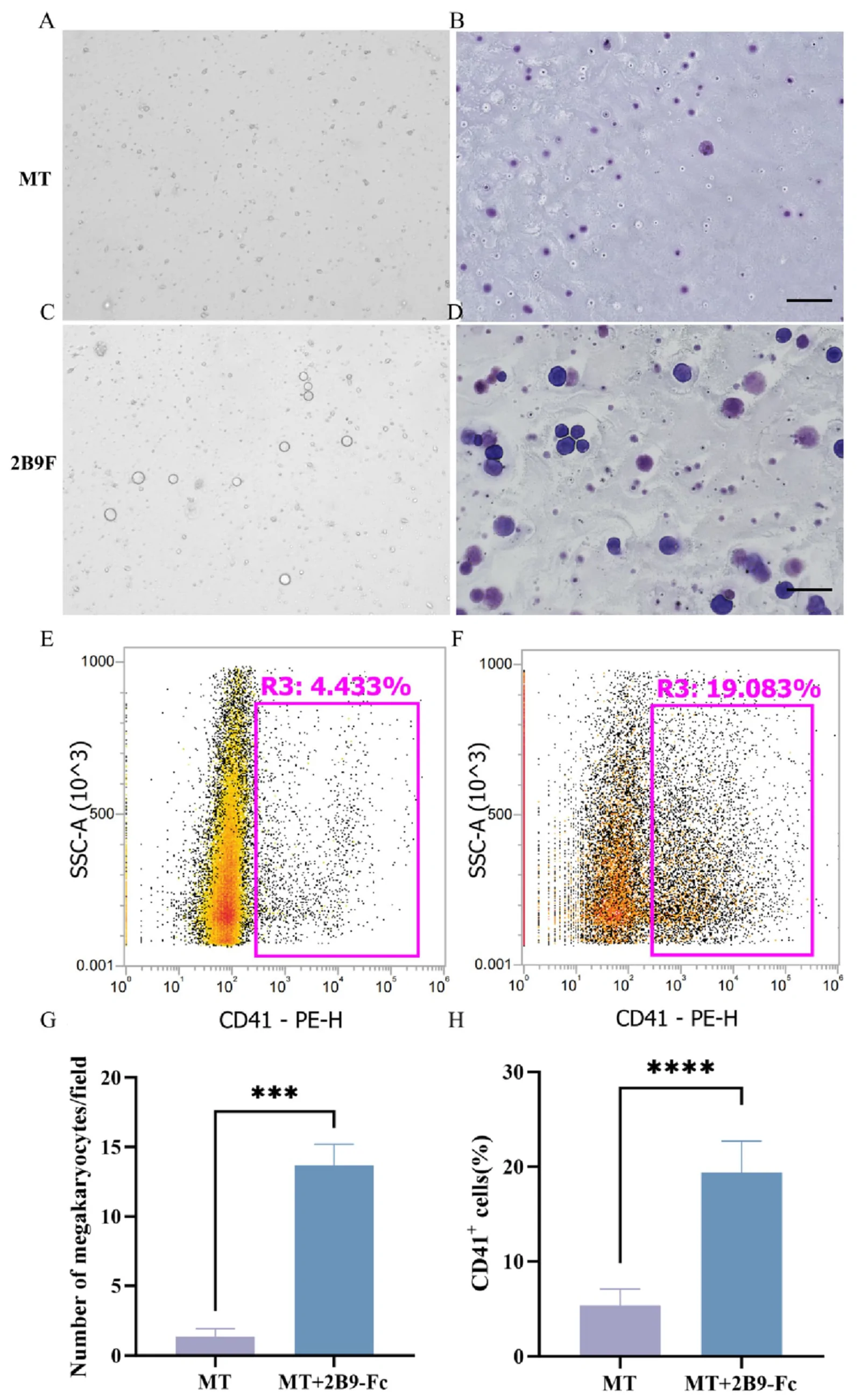
In vitro analysis of 2B9-Fc-mediated activation of the humanized c-MPL receptor. (**A**,**C**) Bright-field microscopy of primary bone marrow cells from MT mice cultured with PBS (**A**) or 2B9-Fc (**C**). (**B**,**D**) Giemsa staining of bone marrow cells cultured with PBS (**B**) or 2B9-Fc (**D**). (**E**,**F**) Representative flow cytometry plots of CD41^+^ megakaryocytes in the PBS (**E**) and 2B9-Fc (**F**) groups. (**G**) Quantification of megakaryocytes per field in Giemsa-stained slides (three randomly selected fields per group). (**H**) Quantification of CD41^+^ megakaryocytes by flow cytometry (three independent experiments). Data are mean ± SD. *** *p* < 0.001, **** *p* < 0.0001. Scale bar, 100 μm.

In vitro, bone marrow mononuclear cells from MT mice were cultured with 2B9-Fc (100 ng/mL) or PBS for 5 days. Bright-field microscopy showed that the 2B9-Fc group contained abundant large cells with abundant cytoplasm and typical lobulated nuclei, whereas the PBS group showed few and immature megakaryocytes (Figure 6A,C). Giemsa staining confirmed these morphological differences: 2B9-Fc-treated cultures contained more mature megakaryocytes with multilobulated nuclei and purplish-red cytoplasmic granules (Figure 6B,D). Quantification of Giemsa-stained slides showed that the number of megakaryocytes per field was significantly higher in the 2B9-Fc group than in the PBS group (mean ± SD: 13.67 ± 1.53 vs. 1.33 ± 0.58, *p* < 0.01; Figure 6G). Flow cytometry showed that the proportion of CD41^+^ megakaryocytes was significantly increased in the 2B9-Fc group compared with the PBS group (mean ± SD: 19.40% ± 3.31% vs. 5.36% ± 1.72%, *p* < 0.001; Figure 6E,F,H). These results indicate that 2B9-Fc efficiently activates the humanized c-MPL receptor and promotes megakaryocyte lineage proliferation and maturation.

**Figure 7:**
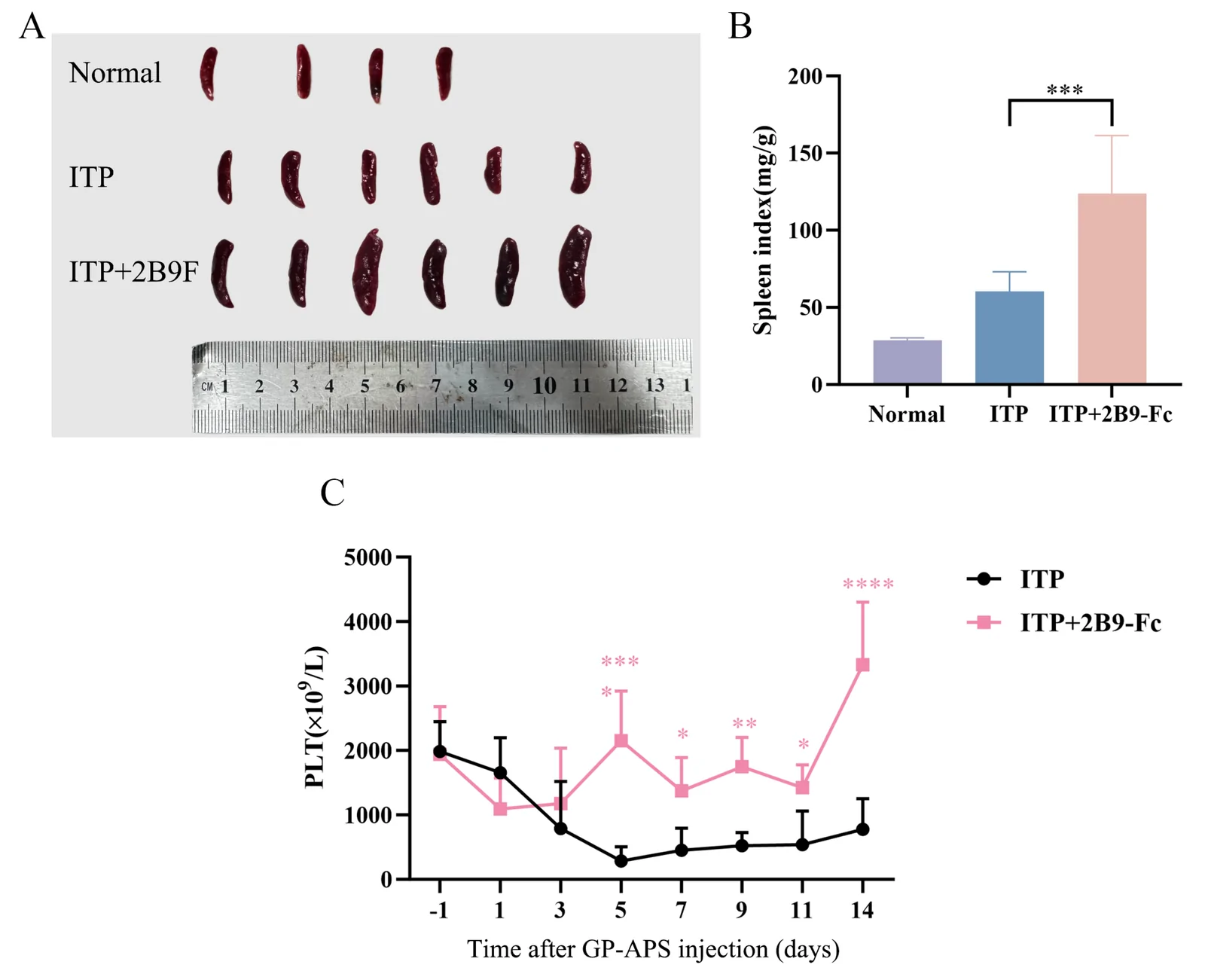
In vivo analysis of 2B9-Fc-mediated activation of the humanized c-MPL receptor. (**A**) Spleen morphology of normal, ITP, and ITP + 2B9-Fc mice at day 14 after modeling. Only the ITP + 2B9-Fc group received 2B9-Fc; (**B**) spleen index of each group 14 days after 2B9-Fc administration; (**C**) changes in PLT in the active immunization ITP model of MT mice after 2B9-Fc administration. n = 6 per group. * *p* < 0.05, ** *p* < 0.01, *** *p* < 0.001, **** *p* < 0.0001.

In the MT active immunization ITP model, PLT in the model group reached a nadir on day 5 after GP-APS injection and stayed low. In the 2B9-Fc group (500 μg/kg, subcutaneously, once every 4 days), the decline was markedly limited: recovery began on day 3, and PLT were significantly higher than in the model group from day 5 onward (*p* < 0.05 or *p* < 0.01). Compared with the wild-type active model, 2B9-Fc maintained platelets at normal levels for longer in MT mice, persisting through day 14 (day 14: 3332 ± 970 vs. 776 ± 474 × 10^9^/L; mean difference 2556 × 10^9^/L, 95% CI 1529 to 3583; Hedges’ g = 3.21), suggesting that the human c-MPL receptor conferred more sustained or more efficient signaling (Figure 7C). The MT-ITP model group showed enlarged spleens and an increased spleen index, and the spleen index increased further after 2B9-Fc treatment. At day 14 after 2B9-Fc administration, histological analysis of paraffin-embedded spleen sections showed that normal spleens had intact white pulp, a normal red/white pulp ratio, and no extramedullary hematopoiesis. In ITP mice, the red pulp was mildly expanded, white pulp was compressed, and scattered megakaryocytes were present, indicating early compensatory extramedullary hematopoiesis. After 2B9-Fc treatment, the red pulp was markedly expanded, white pulp was severely compressed and marginalized, and large clusters of megakaryocytes and hematopoietic precursors were observed, accompanied by sinusoidal dilation and congestion. No necrosis or acute inflammatory infiltration was seen. These histological findings support that the increased spleen index was attributable to extramedullary hematopoiesis rather than lymphoid hyperplasia or acute toxicity (Figure 7A,B and Figure S8). Together, these in vitro and in vivo results show that 2B9-Fc directly and effectively activated the human c-MPL receptor, promoting megakaryocyte maturation and sustained platelet production, and provide key experimental evidence for clinical translation.

## 3. Discussion

ITP treatment has shifted from suppressing platelet destruction alone toward a strategy that simultaneously promotes platelet production and corrects immune imbalance [22,23]. TPO-RAs activate the c-MPL receptor to stimulate megakaryocyte proliferation, differentiation, and platelet release, and they are effective in many patients with refractory ITP. Existing agents nonetheless have limitations: rhTPO is a full-length protein whose long-term use can induce neutralizing antibodies, and small-molecule TPO-RAs show strong species specificity, complicating preclinical evaluation in conventional mouse models [24,25]. TPO mimetic peptides with novel structures, long-acting properties, low immunogenicity, and cross-species activity are therefore of considerable interest. Here we systematically evaluated the previously constructed 2B9-Fc in two ITP mouse models, focusing on megakaryocyte generation and immune regulation. Before the present work, the dose–response profile of this molecule had been characterized in mice during its development for hematopoietic acute radiation syndrome [19]. In that study, 0.5–5 mg/kg was safe and protective, 200 µg/kg was the minimal effective dose for multilineage hematopoietic recovery, and 200 µg/kg compared favorably with romiplostim in a lethal irradiation model. The 500 µg/kg dose used here therefore lies within the safe and effective range and above the minimal effective dose in this species. Because ITP requires repeated administration, the safety margin rather than single-dose peak efficacy was the primary constraint on dose selection. No romiplostim arm was included in the present ITP experiments, so this does not constitute a head-to-head comparison in ITP.

In the passive model, PLT recovered rapidly after a single 2B9-Fc dose, peaked on day 5, and remained significantly higher than in the model group throughout 7 days of persistent antibody exposure. This model recapitulates FcγR-dependent clearance of platelets by splenic macrophages [4,26,27] and therefore demands both sufficient new platelet production and net gain under continuous antibody attack; 2B9-Fc met both demands. Immune profiling showed elevated bone marrow DC, macrophage, and B-cell proportions after ITP induction, an aberrantly activated microenvironment that may interfere with megakaryocyte development and platelet release. These populations declined significantly after 2B9-Fc, with an increased T-cell proportion, suggesting an association with restoration of bone marrow hematopoietic homeostasis. In the spleen, DC proportions decreased and macrophage proportions recovered after treatment, suggesting that 2B9-Fc may also reshape the immune landscape at the primary site of platelet destruction.

In the active immunization model, 2B9-Fc significantly inhibited the GP-APS-induced decline in PLT and markedly alleviated bleeding manifestations such as skin purpura, confirming efficacy under persistent autoantibody attack. Flow cytometry showed an increased splenic M1 proportion, a decreased M2 proportion, and a significantly elevated M1/M2 ratio in the model group [28]; 2B9-Fc treatment was associated with reversal of these changes and a significantly reduced M1/M2 ratio [29,30]. The splenic Treg proportion was already compensatorily elevated in the model group and increased further after 2B9-Fc treatment [20,21]. Thus, 2B9-Fc treatment was associated with M2 macrophage polarization and Treg expansion, and with restoration of splenic immune microenvironment homeostasis [31]. At the transcriptional level, 2B9-Fc downregulated IL-6 and iNOS and upregulated TGF-β1 and Foxp3, consistent with the flow cytometric results. TNF-α showed no difference among groups, whereas Arg-1 was abnormally elevated in the model group and, although reduced by treatment, remained above normal. These patterns are likely explained by cell-type complexity. TNF-α transcription is primarily regulated by the NF-κB pathway rather than STAT1, and whole-spleen qPCR sums transcripts across cell types; megakaryocytes and newly generated platelets stimulated by 2B9-Fc may themselves contribute TNF-α. Arg-1 is also produced by erythroid precursors and myeloid-derived suppressor cells in addition to M2 macrophages, so its elevation in the model group may reflect excessive compensatory extramedullary hematopoiesis in the spleen. Despite this complexity, within the stringently gated macrophage population, the M1 proportion was significantly suppressed and the STAT1-dependent targets IL-6 and iNOS were both downregulated, suggesting anti-inflammatory reprogramming at the macrophage level. Mechanistically, the splenic p-STAT1/STAT1 ratio was significantly elevated in the model group while total STAT1 did not differ, indicating activation mainly at the level of phosphorylation. 2B9-Fc significantly reduced this ratio, closely paralleling the downregulation of IL-6 and iNOS and suggesting that 2B9-Fc may attenuate the core transcriptional program of M1 polarization in association with reduced STAT1 phosphorylation.

The mechanism by which 2B9-Fc suppresses STAT1 phosphorylation remains to be fully defined. c-MPL activation primarily engages JAK2/STAT5 and PI3K/AKT signaling. STAT5 activation may compete with STAT1 for shared receptor docking sites and transcriptional cofactors, thereby limiting STAT1-dependent inflammatory gene expression. In addition, c-MPL signaling may induce SOCS proteins, particularly SOCS1 and SOCS3, which can negatively regulate JAK/STAT1 signaling, and PI3K/AKT activation may attenuate STAT1 phosphorylation. Because 2B9-Fc also expanded Tregs and promoted M2-like polarization in vivo, part of the reduction in splenic p-STAT1 may reflect an indirect effect of the shifted immune microenvironment rather than a direct action on macrophages. The direct mechanism of action of 2B9-Fc involves activation of c-MPL on HSPCs and megakaryocytes. In our previous study in hematopoietic acute radiation syndrome, a single dose of 2B9F expanded functional HSCs, promoted HSC quiescence, reduced apoptosis, and enhanced multilineage reconstitution, with transcriptional changes in Bcl-2, Noxa, p21, and p27 [19]. These effects are consistent with canonical c-MPL downstream signaling, including JAK2/STAT5 and PI3K/AKT. 2B9 was selected by phage display using the murine c-MPL-binding peptide AF12193 as a scaffold and binds both human and mouse c-MPL. TPO mimetic peptides typically engage the membrane-proximal extracellular domain of c-MPL and induce receptor dimerization. Because 2B9 is fused to human IgG1 Fc, 2B9-Fc is bivalent and can present two 2B9 peptides per Fc dimer, which is expected to promote c-MPL dimerization. However, the precise binding epitope, affinity, and stoichiometry were not determined in this study and will require SPR/BLI, mutagenesis, and structural analysis in future work. In the present ITP study, we did not directly measure intracellular signaling in HSPCs; therefore, whether the same signaling events are engaged in the ITP setting remains to be formally demonstrated.

An important consideration is the inflammatory milieu of ITP, in which IFN-γ and other pro-inflammatory cytokines can perturb TPO/c-MPL signaling. IFN-γ has been shown to downregulate c-MPL on HSPCs and impair TPO-mediated signaling, and TPO receptor agonists such as eltrombopag can partially overcome this inhibition and maintain HSPC function under inflammatory conditions [32]. In our study, 2B9-Fc elevated platelet counts and expanded Tregs while suppressing M1 polarization and STAT1 phosphorylation, which may indirectly reduce IFN-γ-driven inflammation in the splenic microenvironment. Whether 2B9-Fc can directly overcome IFN-γ-mediated c-MPL downregulation in HSPCs, analogous to eltrombopag, was not tested in the present study. This remains an important question for future work, particularly in chronic ITP where IFN-γ levels are elevated. The Fc fragment of 2B9-Fc was designed primarily to extend plasma half-life. The dose used here (500 µg/kg) is far lower than IVIG doses required for FcR saturation, and 2B9-Fc directly activated humanized c-MPL and promoted CD41^+^ megakaryocyte generation in vitro, where FcR-mediated clearance is not operative. However, an Fc-silent variant was not tested, so a contribution of FcR-related effects cannot be fully excluded.

Beyond immune regulation, the direct effect of 2B9-Fc on platelet production was verified in humanized c-MPL receptor transgenic mice. In vitro, 2B9-Fc promoted CD41^+^ megakaryocyte generation from MT bone marrow [33]. In the MT-ITP model, PLT recovered from day 3 and persisted through day 14, and the spleen index increased further relative to the model group. Histological analysis supported that the increased spleen index in 2B9-Fc-treated MT mice was attributable to extramedullary hematopoiesis, as evidenced by marked red pulp expansion, megakaryocyte clusters, and hematopoietic precursors, without necrosis or acute inflammation. This is consistent with mobilization of the spleen as a compensatory hematopoietic site. These results provide evidence for direct activation of the human c-MPL receptor by 2B9-Fc and provide key evidence for clinical translation. However, although 2B9-Fc showed promising efficacy in humanized c-MPL knock-in mice, these mice remain mice with a chimeric receptor and cannot fully predict human responses. Before clinical translation, intermediary evidence from other animal species, including non-human primates, will be required to evaluate pharmacokinetics, pharmacodynamics, immunogenicity, and long-term safety. The current findings therefore support further preclinical development but should be interpreted with caution regarding direct clinical application. The therapeutic mechanism may involve a dual-directional action: 2B9-Fc directly activates c-MPL to drive megakaryocyte proliferation, maturation, and platelet release, rapidly replenishing peripheral PLT; in parallel, it blocks M1 polarization by inhibiting STAT1 phosphorylation, reducing platelet destruction in the inflammatory splenic microenvironment, and is associated with Treg expansion and restoration of autoimmune tolerance [34]. Promoting production and preventing destruction thus act synergistically, together constituting the complete therapeutic logic of 2B9-Fc in ITP.

Despite these promising findings, several limitations should be acknowledged. Dose optimization was not performed in either ITP model: a single dose level (500 μg/kg) was evaluated and no head-to-head comparison with an approved TPO receptor agonist was included, so whether this dose is optimal for ITP remains to be established. Because peptide-Fc molecules differ across species in receptor affinity and pharmacokinetics, allometric scaling from the murine doses used here to anticipated clinical doses has not been performed. The mechanistic evidence presented here is largely correlative and based on phenotypic observations; the upstream signaling cascades (e.g., JAK2/STAT5, PI3K/AKT) through which 2B9-Fc modulates macrophage polarization and Treg differentiation remain to be fully elucidated. The mechanism by which 2B9-Fc suppresses STAT1 phosphorylation was not directly explored. We did not perform BMDM experiments, STAT1 knockout, or pharmacological inhibition; therefore, whether the effect is cell-intrinsic or indirect remains unknown. In addition, we did not use a chronic ITP model. The passive and active immunization models are acute or subacute and cannot fully recapitulate the complex, long-term immune dysregulation of chronic ITP. This is a limitation of the present study. Finally, the pharmacodynamic profile and safety of 2B9-Fc have yet to be evaluated in non-human primates. Further investigations are warranted to address these gaps.

## 4. Materials and Methods

### 4.1. Materials

2B9-Fc was obtained by phage-display screening in our laboratory, expressed in ExpiCHO cells, and purified by Protein A affinity chromatography; protein concentration was measured by the BCA method, and the protein was aliquoted and stored at −80 °C [19]. The anti-mouse CD41 monoclonal antibody R300 was purchased from EMFRET (Eibelstadt, Germany) and used for passive immunization modeling and flow cytometric detection. Guinea pig anti-mouse platelet serum (GP-APS) was obtained from Daixuan Biotechnology (Shanghai, China). RPMI-1640 medium and fetal bovine serum (FBS) were purchased from Gibco (Waltham, MA, USA). Flow cytometric antibodies, including CD41-APC, CD4-FITC, CD25-APC, Foxp3-PE, CD11c-APC, MHC II-PE, CD11b-PerCP-Cy5.5, F4/80-FITC, CD86-PE, and CD206-APC, were purchased from BD Biosciences (San Jose, CA, USA) or BioLegend (San Diego, CA, USA).

Male specific pathogen-free (SPF) C57BL/6J mice (6–8 weeks old, 21–23 g) and male humanized c-mpl receptor transgenic (MT) mice were purchased from Huafukang Bioscience (Beijing, China) and Cyagen Biosciences (Suzhou, China), respectively. MT mice were generated by gene replacement at the endogenous Mpl locus. The humanized sequence was confirmed by PCR and sequencing. All MT mice used in this study were homozygous for the knock-in allele. Mice were housed in an SPF facility at 18–24 °C and 40–70% relative humidity under a 12 h light/dark cycle with free access to food and water; cages were provided with paper tubes as environmental enrichment. All mice were acclimatised for 3 days before the start of experiments. All animal experiments were approved by the Institutional Animal Care and Use Committee of the Academy of Military Medical Sciences (approval no. DWZX-IACUC-2026-P627) and followed the ARRIVE 2.0 guidelines. Humane endpoints were predefined as diffuse skin ecchymosis accompanied by active bleeding, body weight loss ≥ 20%, body condition score (BCS) ≤ 2, or respiratory distress/recumbency; animals were monitored once daily and euthanised immediately upon meeting any criterion. Skin purpura alone was not used as a humane endpoint, because it is an expected manifestation of the ITP model and served as an efficacy readout. No anaesthesia or analgesia was administered, as blood sampling and drug administration were brief and minimally invasive; mice were euthanised by cervical dislocation. No unexpected adverse events occurred during the study.

### 4.2. ITP Animal Model Establishment, Grouping, and Treatment

(1) Passive immunization model: mice received subcutaneous injections of the anti-CD41 monoclonal antibody R300 at 0.08 mg/kg every 3 days. Modeling was considered successful when the tail-vein PLT count measured 24 h later fell below 50% of the baseline value. (2) Active immunization model: as described previously [35], platelets from C57BL/6J mice were mixed with complete/incomplete Freund’s adjuvant to immunize guinea pigs for GP-APS production; mice then received a single intraperitoneal injection of GP-APS (100 μL/20 g).

A total of 66 mice were used in this study, with n = 6 per group (Figure 1 and Figure 2, passive immunization model, normal control/ITP model/2B9-Fc groups, 18 mice; Figure 3, wild-type active immunization model, ITP model/2B9-Fc groups, 12 mice; Figure 4 and Figure 5, wild-type active immunization model, normal control/ITP model/2B9-Fc groups, 18 mice; Figure 6, 6 MT donor mice, 2 per independent experiment; Figure 7, MT active immunization model, 12 mice). Mice were randomly allocated to groups using a computer-generated random sequence stratified by body weight. Inclusion criteria were male sex, age 6–8 weeks, body weight 21–23 g and SPF health status; successful modelling was defined as a peripheral platelet count below 50% of the baseline value in both models. No a priori sample size calculation was performed; group sizes were based on our previous experience with the same ITP models. To minimise potential confounders, animals of different groups were housed in separate cages within the same room, and the order of drug administration and measurement was kept constant across groups. No animals or data points were excluded from the analysis. 2B9-Fc was administered subcutaneously at a single dose level of 500 μg/kg throughout the study, and the normal control and model groups received an equal volume of normal saline. In the passive model, 2B9-Fc was given from day 1 after successful modeling and then every 3 days; in the active model, it was given on days 0, 4, 8, and 12.

### 4.3. Peripheral Blood Cell Counting and Bleeding Symptom Assessment

Group allocation and drug administration were performed by the same investigator, who was aware of group allocation. Blood sampling, haematological measurement, skin purpura assessment and data analysis were performed by other investigators who were blinded to group allocation; all samples were labelled with codes rather than group names. The primary outcome measure was the peripheral platelet count. In the passive model, 20 μL blood samples were collected from the tail vein before modeling (day 0) and on days 1, 2, 3, 4, 5, 7, and 10; PLT, WBC, RBC, and HGB were measured with an automated hematology analyzer (MEK-7222K, Nihon Kohden, Tokyo, Japan). In the active model, blood was sampled every other day, and at the endpoint skin purpura was photographed and the spleen index determined.

### 4.4. Flow Cytometric Analysis of Immune Cells

Mice were euthanized and spleens collected aseptically; single-cell suspensions were prepared by grinding and filtration and resuspended in PBS after red blood cell lysis. Cells (1 × 10^6^/tube) were stained as follows: (1) macrophage polarization: surface CD45, F4/80, CD11b, and CD86, then intracellular CD206 after permeabilization; macrophages were gated as F4/80^+^CD11b^+^ cells and analyzed for CD86^+^ M1 and CD206^+^ M2 proportions; (2) DCs: CD11c + MHC II+ cells; (3) T cells and Tregs: surface CD3, CD4, and CD8, with CD4^+^Foxp3^+^ cells defined as Tregs; (4) B lymphocytes: APC-Cy7-conjugated anti-mouse B220 (clone RA3-6B2), with CD45 + B220+ cells defined as B cells (Figures S1–S4). Fluorescence-minus-one (FMO) and isotype controls were used to set gates and exclude nonspecific binding. LSK (Lin^−^Sca-1^+^c-Kit^+^) cells and megakaryocytic lineage cells (Lin^−^CD41^+^) were sorted from bone marrow of WT and MT mice by flow cytometry. Mpl mRNA expression was measured by qPCR with Gapdh as internal control.

### 4.5. RNA Extraction and qPCR

Splenic tissue (~50 mg) from each mouse was homogenized in Trizol reagent using a tissue homogenizer, and total RNA was extracted by the conventional chloroform–isopropanol method. RNA concentration and purity were determined by spectrophotometry (A260/A280 ratio between 1.8 and 2.0). Total RNA (1 μg) was reverse-transcribed into cDNA using the Evo M-MLV RT Premix kit (Accurate Biotechnology, Guangzhou, China). qPCR was performed with 2×SYBR Green Pro Taq HS Premix (Accurate Biotechnology) on a LightCycler^®^ 96 real-time PCR system (Roche, Basel, Switzerland). The thermal cycling conditions were as follows: initial denaturation at 95 °C for 30 s, followed by 40 cycles of 95 °C for 5 s and 60 °C for 30 s, and a final melting-curve analysis. Gapdh was used as the internal reference gene, and relative gene expression was calculated using the 2^(−ΔΔCt)^ method. Primers were synthesized by Sangon Biotech (Shanghai, China) Co., Ltd., and the sequences are listed in Table 1.

### 4.6. H&E Staining

Spleens were fixed in 4% paraformaldehyde, embedded in paraffin, sectioned at 4 μm, deparaffinized, rehydrated, and stained with hematoxylin and eosin (H&E). Sections were examined under a light microscope for red and white pulp structure, megakaryocytes, hematopoietic precursors, sinusoidal dilation, congestion, necrosis, and inflammatory infiltration.

### 4.7. Western Blotting

Splenic tissue (~50 mg) from each mouse was homogenized in RIPA lysis buffer (Beyotime, Shanghai, China) containing protease and phosphatase inhibitors (Roche), lysed on ice for 30 min, and centrifuged at 12,000× *g* for 15 min at 4 °C; the supernatant was collected. Protein concentration was determined by the BCA method (KeyGen Biotech, Nanjing, China). Equal amounts of protein (30–50 μg/lane) were separated by 10% SDS-PAGE and wet-transferred onto PVDF membranes (Millipore, Burlington, MA, USA). After blocking with 5% BSA/TBST for 1 h at room temperature, the membranes were incubated overnight at 4 °C with rabbit anti-mouse p-STAT1 (Tyr701) antibody (1:1000, Cell Signaling Technology, #9167, Danvers, MA, USA), rabbit anti-mouse STAT1 antibody (1:1000, Proteintech, San Diego, CA, USA), and rabbit anti-mouse Actin antibody (1:5000, BioLegend). After washing, the membranes were incubated with HRP-conjugated goat anti-rabbit secondary antibody (1:5000, Abiowell, Changsha, China) for 1 h at room temperature. Signals were developed by enhanced chemiluminescence (ECL) and captured with a Tanon imaging system. Band intensities were quantified with ImageJ software (version 1.54), and the p-STAT1/STAT1 ratio was used to represent the level of STAT1 phosphorylation.

### 4.8. In Vitro Megakaryocyte Generation Assay Using Humanized c-MPL Receptor Transgenic Mice

Bone marrow cells were flushed from the femurs of MT mice to obtain bone marrow mononuclear cells, which were resuspended in RPMI-1640 medium containing 10% FBS or ExpiCHO serum-free medium, seeded in 24-well plates, and cultured with 2B9-Fc (100 ng/mL) or an equal volume of PBS (control) at 37 °C in 5% CO_2_ for 5 days. On day 5, cells were harvested; a portion was used for Giemsa staining to examine megakaryocyte morphology, and the remainder was stained with CD41-APC for flow cytometric determination of the proportion of CD41^+^ megakaryocytes. In addition, MT mice and wild-type C57BL/6J mice were injected subcutaneously with 2B9-Fc at 500 μg/kg, and blood samples were collected on days 0, 1, 3, 5, 7, and 10 for the determination of PLT, WBC, RBC, and HGB. Bone marrow mononuclear cells were obtained from 2 MT mice, pooled, and seeded into 24-well plates; 2B9-Fc (100 ng/mL) or an equal volume of PBS was added to separate wells. Each group comprised three replicate wells, and the experimental unit was the culture well. The experiment was performed three times independently. CD41^+^ megakaryocytes were analysed by flow cytometry using three wells per group.

### 4.9. Giemsa Staining

Bone marrow cells harvested on day 5 of culture were gently resuspended, and an appropriate volume of cell suspension (approximately 30–50 μL) was dropped onto clean glass slides and smeared by the push-slide method, followed by air-drying at room temperature. The smears were fixed with methanol covering the entire specimen area for 2–3 min. After removal of methanol and air-drying, a Giemsa working solution was prepared by diluting Giemsa stain with phosphate-buffered saline (PBS, pH 6.8) at a ratio of 1:9. The smears were placed on a staining rack, covered completely with the Giemsa working solution, and stained at room temperature for 15–30 min. After staining, the slides were rinsed slowly from one end with distilled or deionized water to remove excess stain and air-dried at room temperature. The smears were observed and photographed under a light microscope; megakaryocytes were identified by their large cell bodies, abundant cytoplasm, lobulated or multilobulated nuclei, and purplish-red cytoplasmic granules.

### 4.10. Statistical Analysis

Statistical analysis was performed with GraphPad Prism 8.0 software. Measurement data are expressed as the mean ± standard deviation ($\bar{X}$ ± s). Comparisons among multiple groups were performed by one-way analysis of variance (ANOVA) followed by Tukey’s post hoc test; repeated-measures data were analyzed by two-way repeated-measures ANOVA. *p* < 0.05 was considered statistically significant. Normality was assessed by the Shapiro–Wilk test and homogeneity of variance by Levene’s test. When these assumptions were met, parametric tests were used; otherwise, the corresponding non-parametric test was applied. Effect sizes are reported as Hedges’ g (the small-sample-corrected form of Cohen’s d) with 95% confidence intervals for pairwise comparisons and as partial η^2^ for the ANOVA models, together with 95% confidence intervals for mean differences. Multiple comparisons among groups were performed with Tukey’s post hoc test, which controls the family-wise error rate. For the qPCR data (Figure 5), *p* values were additionally adjusted across the six genes using the Benjamini–Hochberg false discovery rate (FDR) procedure; both unadjusted and FDR-adjusted values are reported. Statistical significance was set at *p* < 0.05.

## 5. Conclusions

Collectively, these findings demonstrate that 2B9-Fc, a novel TPO mimetic peptide, exerts significant therapeutic effects in both passive and active immunization ITP mouse models, elevating peripheral PLT counts and improving bleeding symptoms and splenic pathological injury. The mechanisms may involve promotion of megakaryocyte maturation and are associated with suppression of macrophage M1 polarization, Treg expansion, and restoration of immune tolerance. 2B9-Fc also activates the human c-MPL receptor and shows sustained thrombopoietic activity in MT mice, supporting its potential as a next-generation TPO receptor agonist for clinical translation.

## Figures and Tables

**Table 1:** Primer sequences used for qPCR.

Gene	Primer Sequence (5′→3′)
*Arg-1*	F: CACAGTCTGGCAGTTGGAAGC; R: TGGTTGTCAGGGGAGTGTTG
*Foxp3*	F: CCCATCCCCAGGAGTCTTG; R: ACCATGACTAGGGGCACTGTA
*Gapdh*	F: AGGTCGGTGTGAACGGATTTG; R: GGGGTCGTTGATGGCAACA
*iNOS*	F: ACTCAGCCAAGCCCTCA; R: CTCTGCCTATCCGTCTCGT
*IL-6*	F: AGCCCACCAAGAACGATAG; R: GGTTGTCACCAGCATCAGT
*TGF-β1*	F: ACTGGAGTTGTACGGCAGTG; R: GGGGCTGATCCCGTTGATT
*TNF-α*	F: AGGCACTCCCCCAAAAGATG; R: TGAGGGTCTGGGCCATAGAA

## Data Availability

The original contributions presented in the study are included in the article/Supplementary Material, further inquiries can be directed to the corresponding authors.

## Supplementary Materials

The following supporting information can be downloaded at https://www.mdpi.com/article/10.3390/ijms27219658/s1.
