# Supplementary material for: 2B9-Fc Ameliorates Immune Thrombocytopenia by Suppressing STAT1-Mediated M1 Macrophage Polarization and Expanding Regulatory T Cells
Source: Int J Mol Sci. 2026 Oct 28;27(21):9658. doi: 10.3390/ijms27219658 (PMC13762883; doi:10.3390/ijms27219658)
Supplement: Supplementary file 1 [file ijms-27-09658-s001.zip › ijms-4614575-supplementary.pdf]

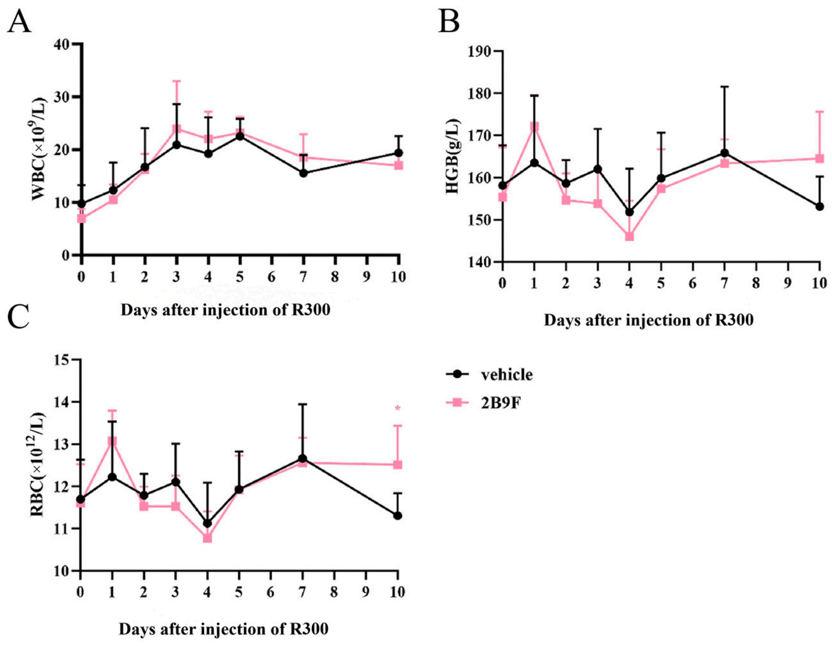

Figure S1. WBC, RBC, and HGB in the passive immunization ITP mouse model.

(A) WBC, (B) HGB, and (C) RBC. No significant differences were observed between the vehicle and 2B9-Fc groups ($P > 0.05$). $n = 6$ per group.

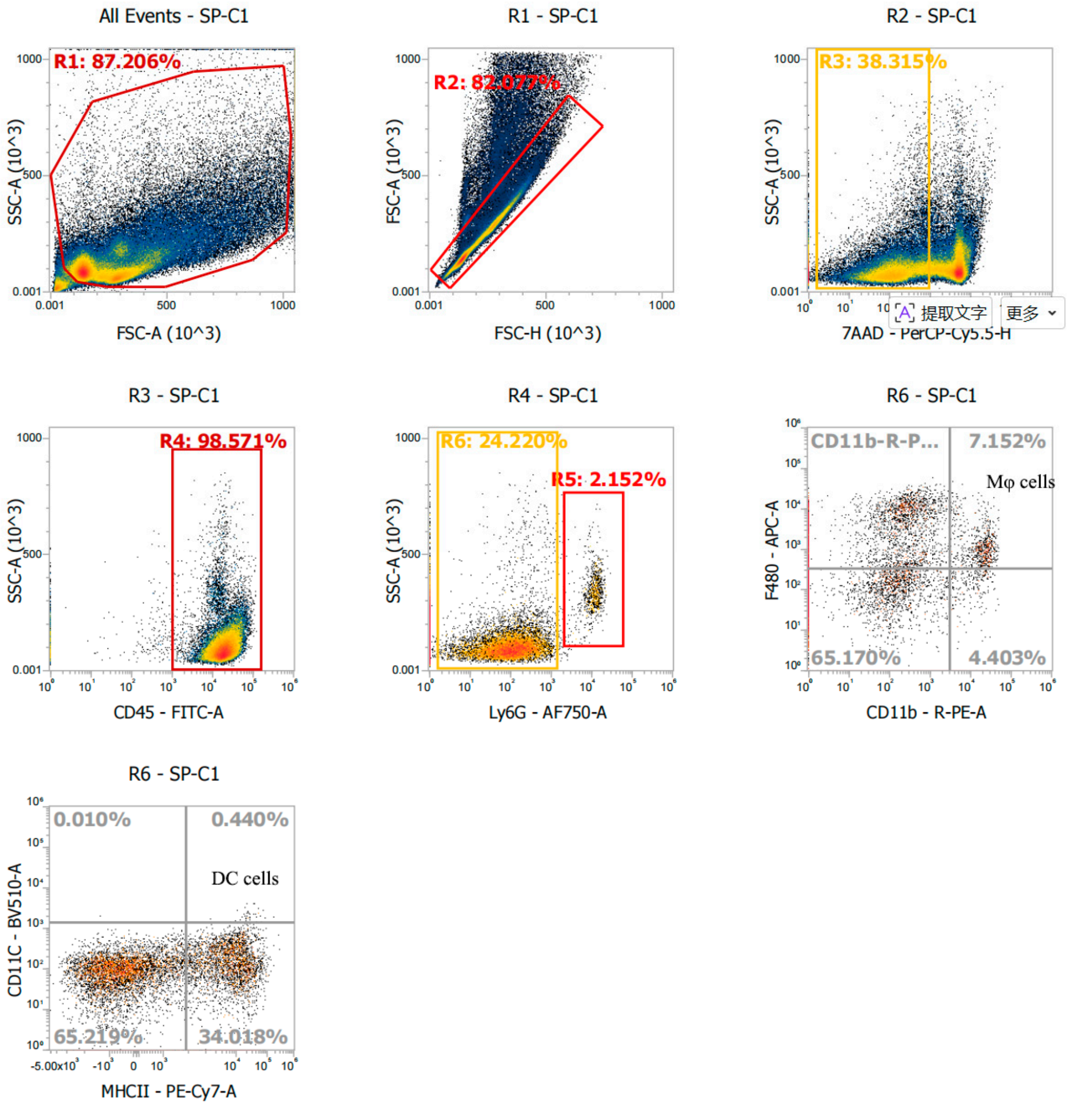

Figure S2. Gating Strategy for Macrophages and Dendritic Cells (DCs)

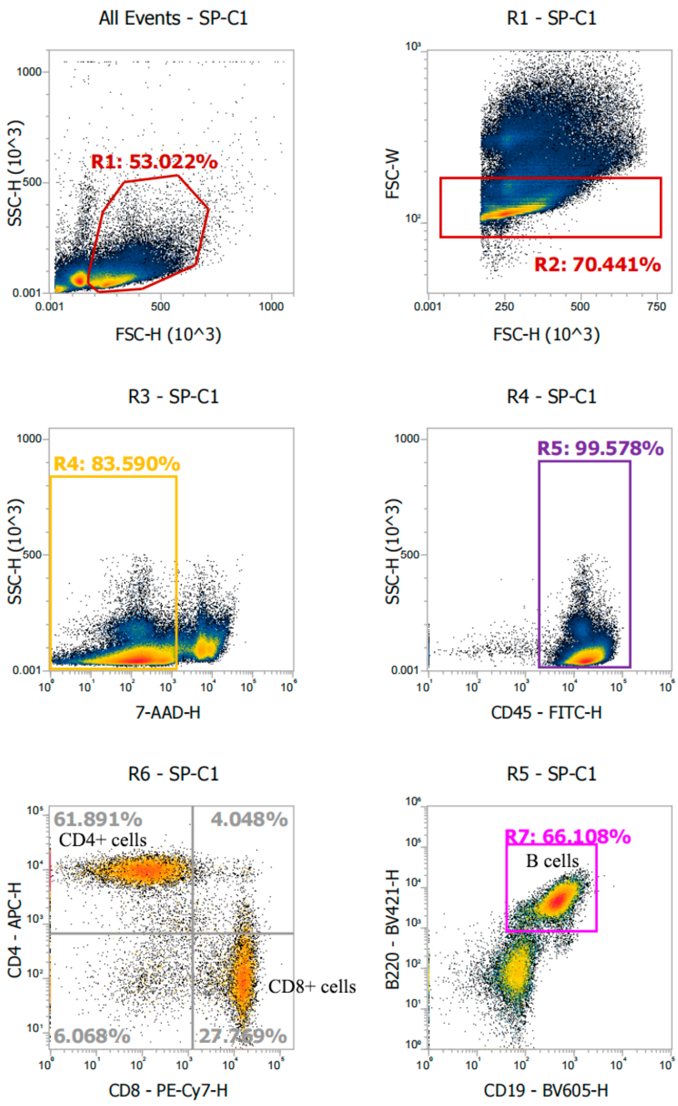

Figure S3. Gating Strategy for T cells and B Cells

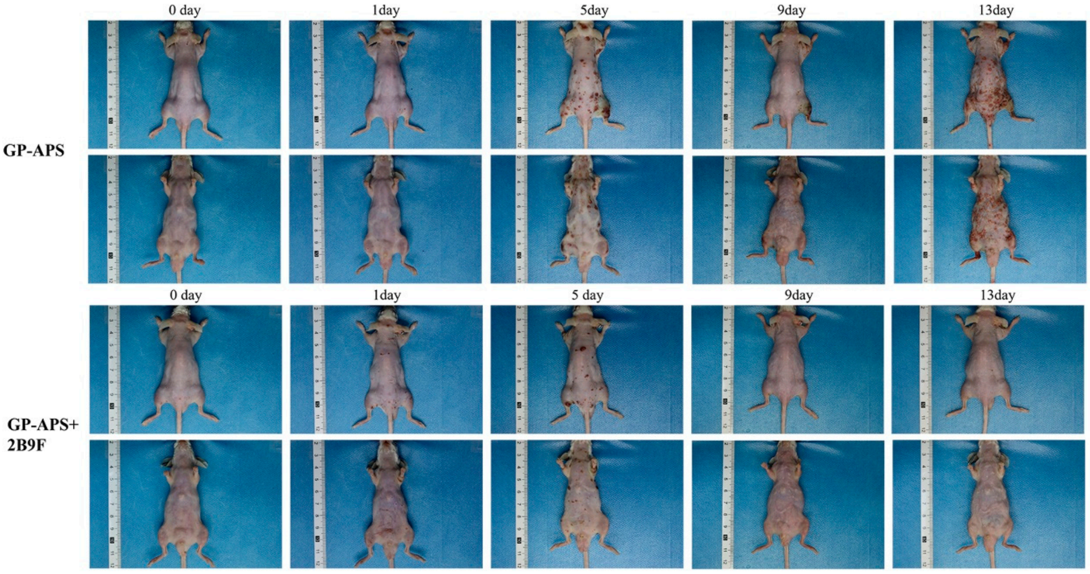

Figure S4. Representative images of skin purpura in GP-APS and GP-APS+2B9-Fc mice at days 0, 1, 5, 9, and 13. n = 6 per group.

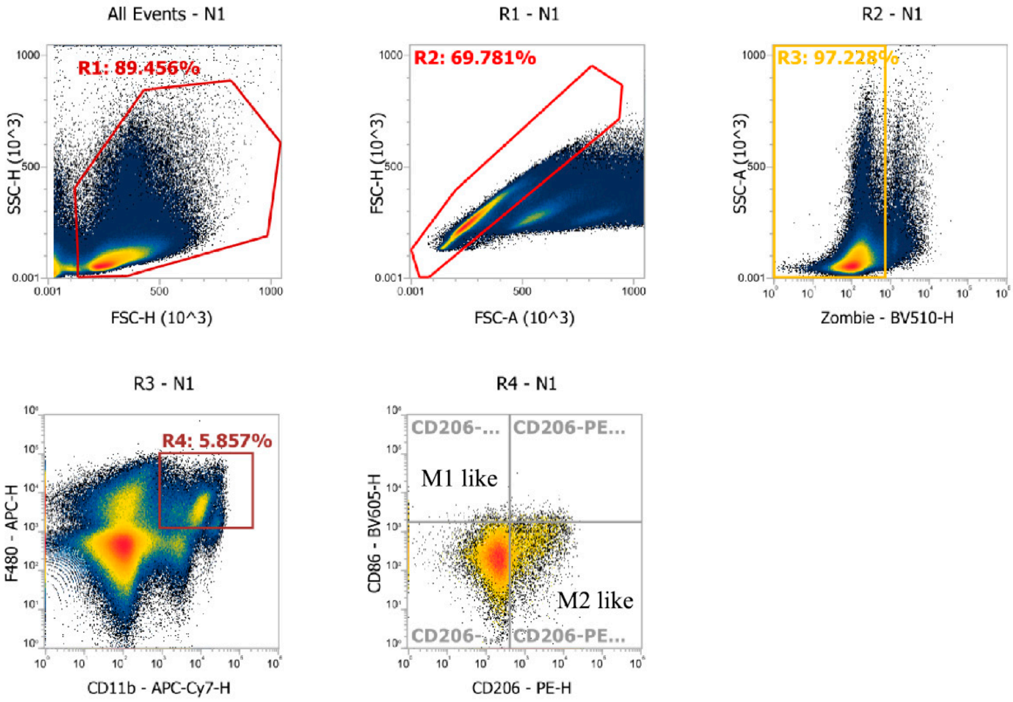

Figure S5. Gating Strategy for M1 and M2 Macrophages.

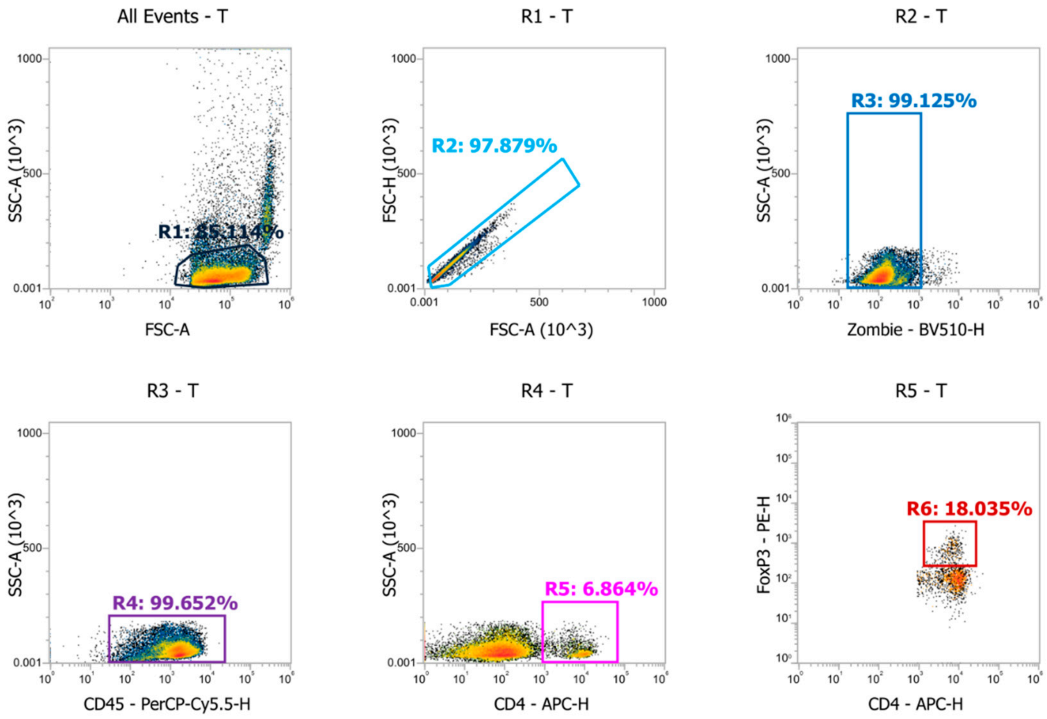

Figure S6. Gating Strategy for Tregs.

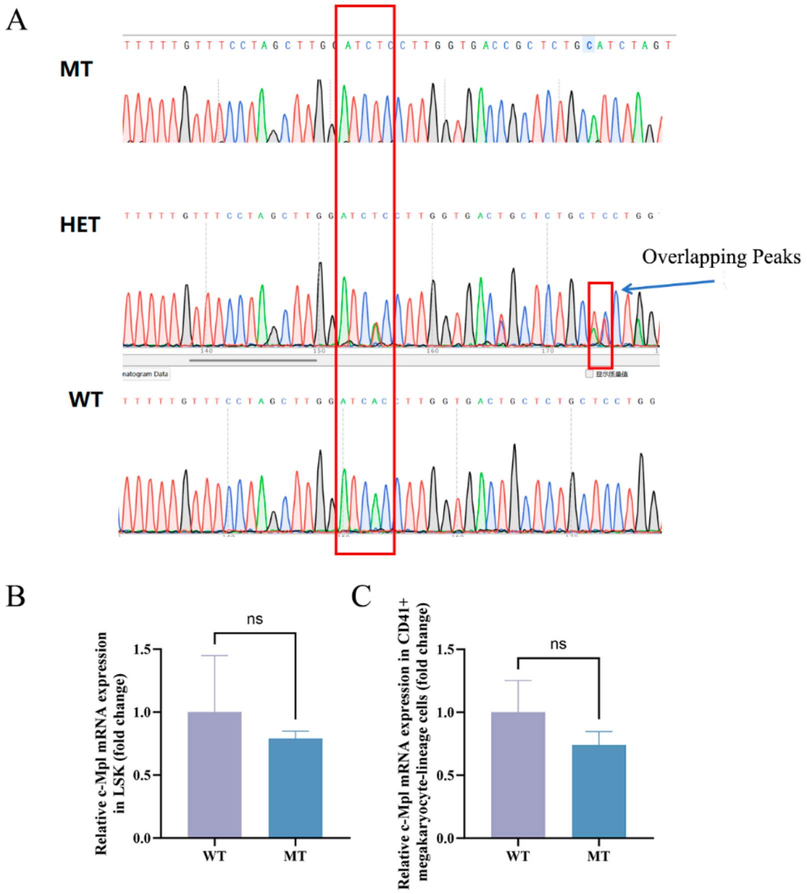

Figure S7. Genetic characterization and Mpl expression in WT and MT mice.

(A) Representative Sanger sequencing chromatograms of the Mpl locus from WT, heterozygous, and homozygous MT mice. The humanized sequence was confirmed in homozygous MT mice.(B, C) Relative Mpl mRNA expression in LSK ($Lin^{-}Sca\text{-}1^{+}c\text{-}Kit^{+}$) cells (B) and megakaryocytic lineage cells ($Lin^{-}CD41^{+}$) (C) sorted from bone marrow of WT and MT mice. Mpl mRNA was normalized to Gapdh. Data are mean ± SD (n = 3 per group). No significant difference was observed between WT and MT mice in either cell population ($P > 0.05$, unpaired two-tailed t test).

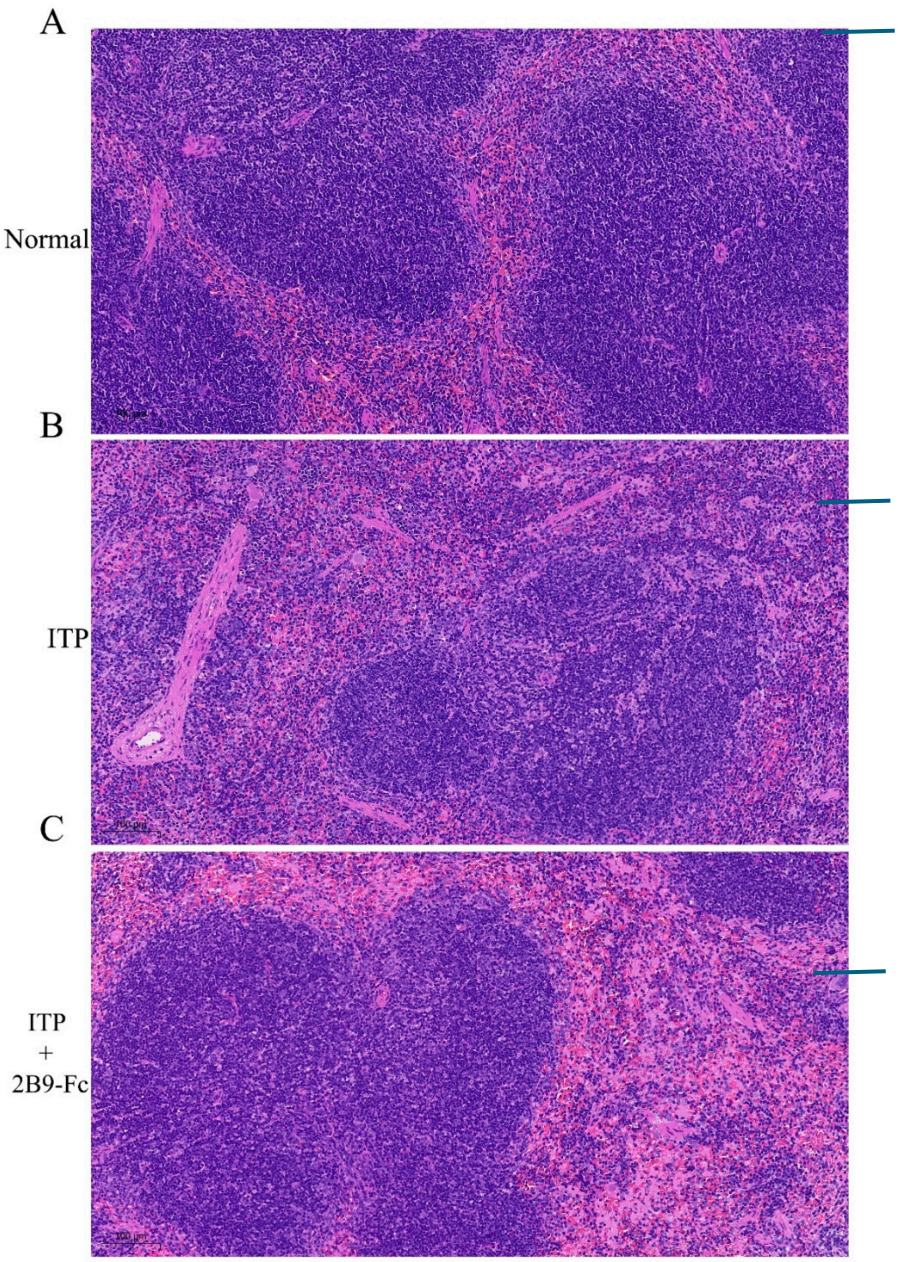

Figure S8. Histological analysis of spleens from MT mice at day 14 after 2B9-Fc administration.

Representative H&E-stained paraffin sections of spleens from normal, ITP, and ITP+2B9-Fc MT mice. Normal spleens show intact white pulp and no extramedullary hematopoiesis. ITP spleens show mild red pulp expansion, white pulp compression, and scattered megakaryocytes. 2B9-Fc-treated spleens show marked red pulp expansion, severe white pulp compression, large clusters of megakaryocytes and hematopoietic precursors, and sinusoidal congestion, without necrosis or acute inflammation. Scale bar, 100 µm. n = 6 per group.
